# Therapeutic Drug Monitoring of Tyrosine Kinase Inhibitors in the Treatment of Advanced Renal Cancer

**DOI:** 10.3390/cancers15010313

**Published:** 2023-01-03

**Authors:** Florent Puisset, Mourad Mseddi, Loïc Mourey, Damien Pouessel, Benoit Blanchet, Etienne Chatelut, Christine Chevreau

**Affiliations:** 1 Institut Claudius-Regaud, Institut Universitaire du Cancer de Toulouse–Oncopole, 31059 Toulouse, France; 2 CRCT, Cancer Research Center of Toulouse, Inserm U1037, Université Paul Sabatier, 31037 Toulouse, France; 3 Department of Pharmacokinetics and Pharmacochemistry, Cochin University Hospital, Assistance Publique-Hôpitaux de Paris, CARPEM, 75014 Paris, France; 4 UMR8038 CNRS, U1268 INSERM, Faculté de Pharmacie, Université Paris Cité, PRES Sorbonne Paris Cité, CARPEM, 75006 Paris, France

**Keywords:** therapeutic drug monitoring, tyrosine kinase inhibitors, renal cancer

## Abstract

**Simple Summary:**

The rational, benefits and limits of therapeutic drug monitoring as a routine practice are discussed for the seven tyrosine kinase inhibitor compounds currently used to treat advanced renal cell carcinoma.

**Abstract:**

Seven tyrosine kinase inhibitor compounds with anti-angiogenic properties remain key drugs to treat advanced renal cell carcinoma. There is a strong rationale to develop therapeutic drug monitoring for these drugs. General considerations of such monitoring of the several groups of anticancer drugs are given, with a focus on oral therapy. Pharmacokinetics and the factors of inter- and intraindividual variabilities of these tyrosine kinase inhibitors are described together with an exhaustive presentation of their pharmacokinetic/pharmacodynamic relationships. The latter was observed in studies where every patient was treated with the same dose, and the results of several prospective studies based on dose individualization support the practice of increasing individual dosage in case of low observed plasma drug concentrations. Finally, the benefits and limits of therapeutic drug monitoring as a routine practice are discussed.

## 1. Introduction

Seven tyrosine kinase inhibitor (TKI) compounds with anti-angiogenic properties associated with their affinity for the vascular endothelial growth factor receptor (VEGFR) are currently the key drugs to treat advanced renal cell carcinoma: cabozantinib, axitinib, sorafenib, sunitinib, pazopanib, lenvatinib, and tivozanib [1]. TKI monotherapy represents the first-choice therapy for treating non-clear cell renal cancer, and the first choice as an alternative therapy if immunotherapy is not tolerated or is inapplicable. TKIs are also combined with an immune check-point inhibitor (CPI) such as pembrolizumab, nivolumab, ipilimumab or avelumab [2,3,4,5,6]. In order to achieve optimal plasma exposure to TKIs, therapeutic drug monitoring (TDM) is now an added consideration which consists of determining plasma concentration in a specific patient to eventually adjust her/his dosage. This practice with TKIs was initially developed for imatinib to treat chronic myeloid leukemia. Trough plasma concentrations at steady-state lower than 1 mg/L have been seen to be associated with a higher risk of treatment failure [7]. Given these initial results, along with the strong rationale for TDM of other TKIs (see below), this practice has begun to be widely used for other TKIs in treating solid tumors including advanced renal cancer [8].

## 2. General Considerations for TDM of Anticancer Drugs

To date, TDM is not carried out for most anticancer drugs, unlike for other drugs such as immunosuppressants. Although close relationships have been described for cytotoxics between their area-under-the-plasma concentration vs. time curve (AUC) and their dose-limiting toxicity, solely determining their plasma concentrations would not allow physicians to adjust dosing for an on-going cycle since these drugs are administered on a Day 1 = Day 21 schedule. Thus, in current practice, cytotoxic dosage is only decreased for the following cycle in case of unacceptable toxicity. This is due to the fact that cytotoxic regimens are composed of two or three drugs, and that the AUC of each one requires multiple blood sampling. Implementation of TDM for cytotoxics is thus hampered [9]. In the case of monoclonal antibodies (Mab) regardless of their pharmacological group (targeted therapies or immune check-point inhibitors), TDM is not justified: no clear pharmacokinetic-pharmacodynamic (PK-PD) relationships have been demonstrated since their dosage is associated with plasma concentrations far higher than those required for activity [10].

Oral targeted therapies, however, corresponding to small molecules and, particularly, TKIs, present all the criteria required to consider TDM, i.e., large interindividual pharmacokinetics (PK) but limited intrapatient PK variability, a better relationship between plasma concentration and pharmacodynamics (PD) than between dosage and PD, long-term treatment, and substantial time lapse between the PD endpoint and the moment of measuring plasma concentrations. Each of these prerequisites will considered for the TKIs used to treat metastatic renal cancer (mRC).

## 3. Pharmacokinetics of TKIs and Their Variabilities

### 3.1. Pharmacokinetics

TKIs in general, and particularly the seven compounds used in mRC, all share similar PK pathways (Table 1). All present sufficient oral bioavailability to be taken orally, are mainly metabolized by CYP3A4, and are also substrates of ABC transporters such as ABCB1 (also called P-glycoprotein) and ABCG2 (BCRP). Their partition coefficient and, accordingly, their lipophilicity are high enough to allow for their passive diffusion through cellular membranes. They are highly bound to plasma proteins such as albumin and α1-glycoprotein acid. However, they present certain PK differences such as a lower oral bioavailability (≈20%) for pazopanib due to poor solubility within the digestive tract, and sunitinib and axitinib (both ≈50%) due to a substantial first-pass enteric effect greater than that of tivozanib (≈85%) [11]. Their metabolites are less active than the parent drug and/or are present at much lower plasma concentrations, except for N-desethyl sunitinib, which contributes to VEGFR inhibition [12]. Their mean half-life ranges between 2–5 h for axitinib to ≈100 h for cabozantinib and tivozanib.

### 3.2. Interindividual Pharmacokinetic Variability

The main PK parameter to consider is oral clearance (CL/F). CL corresponds to the elimination capacity of each patient to clear the drug from plasma, itself mainly dependent on hepatocyte CYP3A4 activity for TKIs; F corresponds to oral bioavailability, which may be much lower than 1 for some of these seven TKIs, and in certain circumstances for all (e.g., taking a gastrointestinal protecting agent too close to TKI administration). Indeed, individual plasma exposure between two drug administrations at steady-state (AUC_τ,ss_), the key parameter for PK/PD relationship (see following paragraph), is equal to the administered dose/(CL/F). As for the numerous drugs sharing these PK pathways (liver metabolism via CYP3A4 and efflux via P-gp), interindividual PK variability (IIV) is large, with coefficients of variation for both CL/F and AUC of 50–100% with extreme values of AUC_τ,ss_ differing by a ratio of around 10. The factors involved in this variability are all well-known: liver function, drug–drug interaction (DDI), age, pharmacogenetics, etc. However, their respective impact cannot be foreseen, thus making predicting individual CL/F values impossible. In the end, the best (and only) way to phenotype each patient is to perform TDM [23].

### 3.3. Intrapatient Pharmacokinetic Variability

As expected for any drug, intrapatient PK variability of the seven TKIs is lower than their respective IIV. Moreover certain factors of intrapatient variability such as DDI, i.e., when a DDI perpetrator—a CYP inducer or inihibitor—is prescribed, represents one of the rationales for TDM. Nevertheless, a degree of intrapatient PK variability remains inexplicable and may be responsible for substantial interoccasion PK variability, thus hampering TDM. This is the case for pazopanib: interoccasion variability between 27 and 61% [16,24] has been observed, possibly questioning the representativeness of the observed concentration with respect to actual global exposure.

## 4. Pharmacokinetic–Pharmacodynamic Relationships for TKI in Treating Renal Cancer

### 4.1. Limits of Standard Doses 

Standard doses of TKIs including those used in mRC are associated with widespread pharmacodynamic effects on both efficacy and adverse events. In terms of efficacy, drug switching is applied if the treatment is no longer effective. Recommendations are made by the manufacturing firm or proposed by investigators for each drug in the case of adverse events. For example, for cabozantinib in case of grade 3 or unacceptable grade 2 adverse events with the standard dose of 60 mg/day, it is recommended to discontinue the drug until adverse events become less severe than grade 1, then to resume treatment at a decreased dose (i.e., 40 mg/day, then 20 mg/day in the case of a second adverse event) [25]. For axitinib, a detailed algorithm has been proposed describing the recommended dose as a function of observed hypertension or proteinuria [26]. This need for individual dosing thus confirms the fact that the PD effects of TKIs are dose-dependent and call for discussion of the methodology used to choose their standard dosage. Phase 1 clinical trials of oral targeted therapies are performed similarly to those traditionally applied to conventional cytotoxic anticancer drugs. A limited number of patients are included at several successively increasing dosage levels up to the maximum tolerated dose. However, since the toxic profile of targeted therapies differs from that of cytotoxic chemotherapies with more chronic adverse events, it has been recommended to extend the period of studying toxicity for TKIs usually to two cycles (each one corresponding to a 3-week period) vs. a single cycle for cytotoxics [27]. Moreover, the number of patients treated at the recommended dose for phase 2 (RP2D) trials is usually larger for targeted therapies (at least 12 patients rather than six for cytotoxics). Despite these customizations, a substantial proportion of patients (around 50%) included in phase 3 trials, and later in routine practice, require dose modifications due to drug-related toxicity [28]. Furthermore, these observations are limited to side effects only. When considering PK-PD relationships (detailed below), a proportion of patients who do, in fact, tolerate well the standard dose are under-exposed and could benefit from higher dosage [29].

### 4.2. Pharmacokinetic–Pharmacodynamic Relationships

As explained above, one of the prerequisites for TDM implementation is confirming the existence of a PK/PD relationship with regards to efficacy and/or safety. The most robust clinical endpoints for exposure–response analysis are dose-limiting toxicity (DLT) or toxicity grade ≥ 3 onset for safety; and for efficacy, progression-free survival (PFS) and overall survival (OS). Various PK/PD relationships use different steady-state PK biomarkers such AUC_τ,ss_, peak concentration (C_max,ss_) and trough concentration (C_min,ss_). C_max,ss_ corresponds to the highest drug concentration in the plasma, while C_min,ss_ is the drug concentration just before the next administered dose. Table 2 summarizes the different PK/PD relationships for axitinib, cabozantinib, pazopanib, sorafenib and sunitinib. As far as we know, no exposure–efficacy data are currently available for lenvatinib or tivozanib in mRC, but reports of drug development show a trend in exploring the PK/toxicity relationship. 

The pharmacodynamic effects of pazopanib, axitinib, cabozantinib, and sorafenib are mainly driven by the parent drug, which explains why only plasma exposure to the parent drug has been used as a PK biomarker. In contrast, preclinical experiments have shown that N-desmethyl sunitinib (SU12662) is an active metabolite [12]. Furthermore, its plasma exposure represents around a third of that of the parent drug in humans, suggesting that SU12662 significantly contributes to the PD effect of sunitinib malate. In this context, plasma concentrations of both sunitinib and SU12662 have been considered in PK/PD studies. The main side effect corresponding to a class-effect of VEGFR-TKIs, i.e., hypertension, is frequently correlated with plasma TKI exposure. Whatever the TKI, higher plasma exposure clearly contributes to the onset of DLT or toxicity grade ≥ 3. Furthermore, different threshold values of PK biomarkers (AUC_τ,ss_, C_min,ss_, or C_max,ss_) have been proposed to prevent DLT onset [30,31,32,33,34]. These threshold values could help physicians document whether DTL onset is concentration-dependent (i.e., due to TKI plasma overexposure). In this case, decreasing TKI dosing without compromising the antitumor effect could be proposed. As for efficacy, the PK/PD relationship is less self-evident since the antitumor response is multifactorial. For pazopanib, the target C_min,ss_ > 20 μg/mL from PK/PD data of a phase 2 trial [35] has been confirmed in different studies [32,36,37] and therefore can be used in daily clinical practice. PK/PD data from the phase 3 METEOR trial ^20^ suggest a target for cabozantinib of C_min,ss_ ≥ 750 ng/mL, but other «real-world» studies [32,38,39] have not confirmed this. Furthermore, these studies also showed that patients with C_min,ss_ ≥ 750 ng/mL would be more at risk of developing DLT. Therefore, other studies are warranted to refine target C_min,ss_. For sunitinib, the target C_min,ss_ > 50 ng/mL has been proposed using PK/PD data from preclinical and phase 1 studies [14,40]. Current data from PK/PD studies in mRC patients do not make it possible to draw any conclusion about the robustness of this target C_min,ss_ in this population. Finally, the axitinib target AUC_τ,ss_ ≥ 300 ng/mL∙h from PK/PD data of a phase 2 trial [41] has been confirmed in a single study [42], while no target value can be proposed for C_min,ss_ and C_max,ss_ [33,34]. Overall, it is possible to note that all of these PK-PD relationships were expected, since both efficacy and adverse events are tightly linked with competitive ATP inhibition of TK receptors, particularly of VEGFR. TDM could be useful in daily clinical practice even if the robustness of target concentrations remains questionable for two reasons. First, PK/PD data from phase 2/3 studies include selected patients with a better general health status than that observed in «real-world» patients. Second, the statistical power in several observational studies remains difficult to confirm, and the therapeutic range problematic to refine due to the small sample sizes of these studies.

**Table 2:** Pharmacokinetic/pharmacodynamic relationships of the seven tyrosine kinase inhibitors prescribed in metastatic renal cancer.

	Number of Patients	PK Marker	PD Efficacy	PD Tolerability	Ref.
Sorafenib	52	C_min,ss_		Threshold C_min,ss_ for onset of grade ≥ 2 hand–foot skin reaction and hypertension: 5.78 and 4.78 μg/mL, respectively	[43]
Sunitinib	149	Composite AUC_ss_ (sunitinib+SU12662)	Positive relationship between composite AUC_ss_ probability of response (partial of complete) or stable disease Association between higher composite AUC_ss_ and longer TTP and OS	Negative relationship between cumulated composite AUC_ss_ at day 28 and absolute neutrophil count	[12]
	55	AUC_ss_ Composite AUC_ss_ (sunitinib+SU12662)	Longer median OS in patients with composite AUC_ss_ > 1973 ng/mL∙h: 35.2 (CI 95%, 26.5–ND) vs. 16.7 (CI 95%, 4.3–ND) months; *p* = 0.0051 Trend for longer median PFS in patients with composite AUC_ss_ > 1973 ng/mL∙h: 35.2 (CI 95%, 8.0–ND) vs. 8.4 (CI 95%, 3.7–ND) months; *p* = 0.15	High sunitinib AUC_ss_: independent risk factor of grade ≥ 3 acute toxicity (OR = 1.16 (CI 95%, 1.05–1.28); *p* = 0.005) High SU12662 AUC_ss_: independent risk factor of grade ≥ 2 thrombocytopenia (OR = 1.27 (CI 95%, 1.03–1.57); *p* = 0.028)	[44]
	21	Composite C_min,ss_ (sunitinib+SU12662)	Patients with composite C_min,ss_ < 100 ng/mL vs. those with composite C_min,SS_ >100 ng/mL: Longer median TTF: 590 vs. 71 days, respectively; *p* = 0.04 Longer median PFS: 748 vs. 238 days, respectively; *p* = 0.02 Trend for longer median: 939 vs. 570 days; *p* = 0.07	Positive relationship between increased composite C_min,ss_ and severity of anorexia (*p* < 0.05) and fatigue (*p* < 0.05) Inverse correlation between composite C_min,ss_ and platelet counts (*p* < 0.05) Higher incidence of grade ≥ 3 toxicities in patients with composite C_min,ss_ > 100 ng/mL (75.0% vs. 23.1%, respectively)	[30]
	20	Composite C_min,ss_ (sunitinib+SU12662)	Patients with composite C_min,ss_ < 50 ng/mL vs. those with C_min,ss_ > 50 ng/mL: Longer median TTF: 743 (CI 95%, 217–1583) vs. 56 (CI 95%, 21–179) days; *p* < 0.001 Longer median PFS: 731 (CI 95%, 197–1576) vs. 95 (CI 95%, 197–1576) days; *p* < 0.001	Higher median composite C_min,ss_ within 6 weeks in patients with DLT than in those without: 92.7 (range 52.7–196.9) vs. 43.4 (38.3–54.1) ng/mL; *p* = 0.001	[45]
	63	Composite AUC_ss_ (sunitinib+SU12662)	At disease progression, trend to a lower composite AUC_ss_ than the one during the first cycle: 1678 vs. 2004 ng/mL.h, respectively; *p* = 0.072 Median PFS not statistically longer in patients with composite AUC_ss_ > 2150 ng/mL.h at cycle 1: 14.8 (CI 95%, 2.7–26.9) vs. 11.4 (CI 95%, 5.8–17.0) months; *p* = 0.45	High composite AUC_ss_: independent risk factor of grade ≥ 3 acute toxicity (OR = 2.72 (CI 95%, 1.84–4.02); *p* < 0.0001) → Target composite AUC_ss_ < 2150 ng/mL.h to prevent onset of grade ≥ 3 acute toxicity	[46]
Cabozantinib	319	C_min,SS_	Average C_min,ss_: 375, 750 and 1125 ng/mL for 20-, 40-, and 60-mg day, respectively. HR for risk of progressive disease or death: - 40 vs. 60 mg/day: 1.10 (CI 95%, 1.07–1.12) - 20 vs. 60 mg/day: 1.39 (CI 95%, 1.29–1.49) → Target C_min,SS_ > 750 ng/mL for efficacy	Association between an increase in average C_min,ss_ and increased risk: - palma–plantar erythrodysesthesia syndrome (grade ≥ 1) - fatigue/asthenia (grade ≥ 3) - hypertension (systolic blood pressure > 160 mmHg or diastolic blood pressure > 100 mmHg) - diarrhea (grade ≥ 3)	[20]
	76	C_min,ss_ AUC_ss_ C_max,ss_	Lower median exposure in patients with progressive disease vs. patients with best disease control - C_min,ss_: 406 vs. 634 ng/mL; *p* = 0.001 - AUC_ss_: 16 vs. 20 mg/mL.h; *p* = 0.037 Target C_min,ss_ > 537 ng/mL for efficacy	Higher median exposure in patients with DLT than in those without - C_max,ss_: 732 vs. 531 ng/mL; *p* = 0.006 - AUC_ss_: 21 vs. 16 mg/mL.h; *p* = 0.046 Target C_min,ss_ < 618 ng/mL to prevent onset of DLT	[31]
	25	C_min,SS_	No difference in PFS in patients with C_min,SS_ < 573 ng/mL with others: 19.0 (CI 95%, 0–45.7) vs. 34 (CI 95%, 32.6–35.5) weeks, respectively	Trend for higher median C_min,ss_ in patients with DLT than in those without: 769 ng/mL (CI 95%, 663–893) vs. 568 ng/mL (CI 95%, 384–842), respectively; *p* = 0.079	[39]
	59	C_min,ss_	No statistical difference in PFS in patients with average C_min,SS_ ≥ 750 ng/mL (over the whole treatment period) compared to others: 19 (CI 95%, 0–40) vs. 52 (CI 95%, 34–70) weeks, respectively; *p* = 0.2 Trend for longer PFS in patients with average C_min,SS_ ≤ 572 ng/mL (over the whole treatment period) compared to others: 65 (CI 95%, not reached) vs. 42 (CI 95%, 20–64) weeks, respectively; *p* = 0.055 No statistical difference in OS in patients with average C_min,SS_ ≤ 572 ng/mL (over the whole treatment period) compared to others	Higher median C_min,ss_ in patients with DLT than in those without: 831 (CI 95%, 711–1040) vs. 569 ng/mL (CI 95%, 494–754); *p* = 0.001 Higher DLT incidence in patients with a C_min,ss_ ≥ 750 ng/mL at start dose compared to patients with an exposure < 750 ng/mL (78.6% vs. 38.7%; *p* = 0.003)	[38]
Pazopanib	177	C_min,ss_	Longer median PFS in patients with C_min,ss_ > 20.5 μg/mL at week 4: 52.0 vs. 19.6 weeks, respectively; *p* = 0.00378 Five-fold greater median observed tumour shrinkage in patients with C_min,ss_ > 20.5 μg/mL at week 4: 37.9% vs. 6.9%.	Increased incidence of grade ≥ 3 toxicity in patients with C_min,ss_ in the fourth quartile (36 to 85 μg/mL)	[35]
	35	C_min,ss_	Longer median PFS in patients with C_min,ss_ > 20 μg/mL: 34.1 vs. 12.5 weeks, respectively; *p* = 0.0271; C_min,SS_ > 20 μg/mL: independent protective factor for death, HR 0.25 (CI 95%, 0.076–0.81); *p* = 0.021		[36]
	311	C_min,ss_	Patients achieving early or late C_min,ss_ > 20.5 μg/mL had significantly longer disease-free survival: - Group early C_min,SS:_ not estimable vs. 29.5 months, HR 0.556 (95% CI, 0.337-0.918); *p* = 0.0055 Group late C_min,ss:_ not estimable vs. 29.9 months, HR 0.583 95% CI, 0.369-0.921); *p* = 0.0078	Increased incidence of grade ≥ 3 hypertension according to quartile in group early C_min,ss_	[37]
	27	C_min,SS_	Objective response rate (complete response or partial response) similar in patients with C_min,SS_ between 20.5 to 50.3 μg/mL and those with C_min,SS_ ≥ 50.3 μg/mL (45.5 vs. 46.2%) No objective response observed in patients with C_min,ss_ < 20.5 μg/mL	Positive relationship between increased C_min,ss_ and severity (grade 0–1 vs. grade ≥ 2) of anorexia (*p* < 0.05), fatigue (*p* < 0.05) and hypertension (*p* < 0.05) Higher incidence of grade ≥ 3 toxicity in patients with a C_min,ss_ ≥ 50.3 μg/mL (61.5% vs. 7.1%) → Target C_min,ss_< 50.3 μg/mL to prevent onset of grade ≥ 3 toxicities	[32]
Axitinib	168	AUC_ss_	Longer median PFS in patients with AUC_ss_ ≥ 300 ng/mL∙h: 13.8 vs. 7.4 months; HR 0.558 (95% CI, 0.379–0.823); *p* = 0.003 Longer median OS in patients with AUC_ss_ ≥ 300 ng/mL∙h: 37.4 vs. 15.8 months; HR 0.489 (95% CI, 0.324–0.738); *p* < 0.001	Weak correlation between AUC_ss_ and blood diastolic pressure (r^2^ < 0.10)	[41]
	33	C_min,ss_ AUC_ss_	Longer OS in patients with C_min,ss_ ≥ 5 ng/mL: median not reached vs. 299 days, respectively; *p* = 0.022) Longer OS in patients with AUC_ss_ ≥ 300 ng/mL∙h: median not reached vs. 409 days, respectively; *p* = 0.045) No relationship between PFS and AUC_ss_ or C_min,ss_	Threshold value C_min,ss_: 6.6 and 7.1 ng/mL to predict grade ≥ 2 hypothyroidism (*p* = 0.005) and grade ≥ 2 anorexia (*p* = 0.035), respectively	[42]
	20	C_max,ss_	Higher C_max,ss_ higher in responders (complete or partial response) than in non-responders (stable or progression disease); *p* = 0.013 Longer median PFS in patients with C_max,ss_ > 12.4 ng/mL: 799 (95% CI, 140–not estimable) vs. 336 days (95% CI, 70–not estimable) days; *p* = 0.047	Higher cumulative incidence of DLT in patients with C_max,ss_ ≥ 40.2 ng/mL: sub-hazard ratio, 4.13 (95% CI, 1.27–13.5); *p* = 0.019	[33]
	35	C_min,ss,first_ (2 weeks after treatment start) C_min,ss,1-3m_ (mean C_min,ss_ between 1 and 3 months after treatment start)	Statistical association between best response and plasma exposure (C_min,ss,first_ and C_min,ss,1-3m_) Higher plasma exposure in patients with PFS ≥ 5 months: - C_min,ss,first_: 1.24 vs. 0.52 ng/mL, respectively; *p* = 0.003 - C_min,ss,1-3m_: 1.76 vs. 0.57 ng/mL, respectively; *p* = 0.001 Trend to higher C_min,ss,1-3m_ in patients with OS ≥ 25 months: 1.64 vs. 0.64 ng/mL, respectively; *p* = 0.097	Higher plasma exposure in patients with grade ≥ 3 toxicity: - C_min,ss,first_: 3.17 vs. 0.73 ng/mL, respectively; *p* = 0.012 - C_min,ss,1-3m_: 2.50 vs. 0.73 ng/mL, respectively; *p* = 0.003	[34]
Tivozanib	432	C_average_ over a 4 weeks treatment period		Logistic regression between C_average_ and probability for hand–foot syndrome: OR = 1.2 [95%CI: 1.00–1.03]	[47]
Lenvatinib	260	C_min,ss_		Plasma exposure based on starting dose was a significant predictor for the occurrence of any grade proteinuria, nausea, and vomiting, and for Grade 3 or higher hypertension	[19]

C_min,ss_, trough concentration at steady-state; C_max,ss_, peak concentration at steady-state; C_average_, mean concentration; AUC_ss_, area-under-the-curve between two administrations at steady-state; TTP, time to progression; OS, overall survival; OR, overall response; PFS, progression free survival; ND, not determined; HR, hazard ratio; OR, odds ration; CI, confidence interval.

## 5. Benefit of TDM in Clinical Practice

### 5.1. TDM as a Predictive Factor for Clinical Outcome

Different studies using TDM for dosing adjustments (e.g., for pazopanib, sunitinib) have been conducted in patients with mRC [48,49] or solid tumors [29,50,51]. Herein lies the real benefit of TDM: in the case of observed C_min,ss_ below threshold efficacy in a patient with “too” good tolerance, TDM allows the physician to increase the individual dose to reach optimal exposure. On the contrary (i.e., C_min,ss_ higher than the threshold value together with observation of poor tolerance), TDM results provide other information in addition to clinical observations, allowing the physician to adjust the dosage less empirically. For example, a recent study reported that 14 out of 31 mRC patients (45%) presented a sunitinib C_min,ss_ within the 50–100 ng/mL range [49]. In other patients, C_min,ss_ was below 50 ng/mL in two cases (7%) and > 100 ng/mL in 15 (48%). For patients under- or over-exposed, PK model-based recommendations for tailored dosing ranged from 12.5 to 100 mg, i.e., a −75% to +100% change as compared with initial standard dosing. However, PK model-based recommendations being retrospective, the authors were not able to assess the clinical benefit of TDM. Moreover, the low number of patients in this study limited the scope. Verheijen et al. showed the feasibility of a PK-guided approach for pazopanib (target C_min,ss_ > 20 μg/mL) in 30 mRC patients with repeated blood sampling [48]. Over the treatment course, 17 patients (57%) had at least one C_min,ss_ < 20 μg/mL following the standard daily dose of 800 mg, and 10 of them were successfully treated with a PK-guided dose escalation, leading to daily dosing ranging from 1000 to 1800 mg. These results are particularly noteworthy since the intrapatient PK variability of pazopanib is higher than that of the other TKIs, allowing us to speculate that the possibility of reaching a target concentration would be even greater for the other VEGFR-TKIs.

The recent Dutch Pharmacology Oncology Group Therapeutic Drug Monitoring study (DPOG-TDM) has assessed the feasibility and the benefit of PK-guided dose optimization in 552 cancer patients for those patients underexposed [29]. In evaluating patients treated with sunitinib (n = 50) and pazopanib (n = 49), PK-guided dose interventions were successful in 80% of cases. However, it should be noted that “success” was based only on pharmacokinetic and toxicity criteria (i.e., if the median C_min,ss_ after intervention was above the predefined TDM target concentrations, and if no dose reduction due to toxicity was needed within one month). Due to its non-randomized design, it was not possible to compare efficacy corresponding to TDM intervention with standard treatment. Splitting intake moments is highly recommended to increase drug bioavailability in patients underexposed at the approved dose of pazopanib (800 mg/day). This strategy is related to the saturation of intestinal absorption above an 800 mg-dose [52]. Groenland et al. showed that administering 400 mg twice daily resulted in a 79% increase in C_min,ss_ compared to 800 mg once daily, with acceptable safety [53]. For sorafenib, PK-guided dose interventions were rarely feasible due to toxicity. Nevertheless, it is also important to note that the splitting strategy can be used for sorafenib doses above 400 mg twice daily due to saturation of intestinal absorption [54]. Finally, the benefit of TDM for axitinib could not be assessed since only two patients were included. The main limitation of published TDM studies in mRC patients is the lack of a control group to clearly evaluate the benefit of the PK-guided approach. To this end, a large multicenter randomized controlled trial with PFS or overall survival as an endpoint would be warranted, such as that previously conducted for imatinib in chronic myelogenous leukemia patients [55]. However, the rapid turnover of TKIs, added to their future combination with immunotherapy, prevents pharmacologists from designing and conducting such a trial.

### 5.2. Patients with Poor Adherence

The adherence to oral targeted therapies for cancer has not been formally studied in patients with mRC. However, several studies performed with TKIs in other diseases such as chronic myeloid leukemia showed that an adherence rate below 90% can be seen in nearly 25% of patients [56]. One of the objectives of TDM is to identify poorly adherent patients by observing a low TKI plasma level. However, while plasma concentrations below or just above the quantification limit of the assay may reveal poor adherence, some low concentrations may result from either poor adherence or a high metabolic capacity with no possibility of discriminating between these two situations. This is a key fact since the appropriate recommendation in each case is not the same: for poor adherence, therapeutic education would improve treatment adherence and no dose change would be recommended; for high oral CL, on the contrary, a higher daily dose of TKI should be used. The hypothesis can be raised that simultaneously determining the concentrations of the drug (parent compound) and one (or more) of its metabolites would make it possible to differentiate between these two patient profiles since patients with high metabolic capacity should have relatively high metabolite concentrations, while poorly adherent patients would have very low metabolite concentrations. This hypothesis has never been demonstrated or even evaluated for TKIs, but has been shown for atorvastatin; monitoring the drug and its two metabolites was more successful than monitoring atorvastatin concentrations alone in identifying non-adherent patients [57].

### 5.3. Management of Drug–Drug Interactions

Patients with mRC are usually treated with a large number of co-medications, thereby increasing the risk of DDI [58]. In particular, calcium-channel blockers are often given as antihypertensive drugs to treat this TKI-induced condition [59]. Since some of them are weak or moderate CYP3A inhibitors, clinicians must choose between changing the antihypertensive at the risk of unbalancing blood pressure, or allowing the DDI at the risk of increased toxicity. In the case of well-controlled blood pressure, TDM makes it possible to maintain the antihypertensive drug and adapt the TKI dose only in the case of significant overexposure.

## 6. Clinical Practices and Practical Issues

### 6.1. AUC_τ,ss_ vs. C_min,ss_ as the Best Marker of Systemic Exposure

Several observations corresponding to PK data obtained during the drug’s clinical development confirm that the best marker of drug plasma exposure is AUC_τ,ss_ corresponding to the area-under-the-curve of plasma vs. time concentrations between two administrations at steady-state. This determination requires several blood samplings compatible with a clinical trial methodology but not with routine practice, particularly in an outpatient context. Fortunately, however, close correlations have also been reported between AUC_τ,ss_ and C_min,ss_ with regards to TKI administration schedule that were indeed to be expected; daily administration is associated with limited fluctuations of plasma concentrations, given their relatively long elimination half-life. Furthermore, individual C_min,ss_ values are similar to the corresponding individual C_mean,ss_ values that are equal to AUC_τ,ss_/τ. For all of these reasons, the concept of a C_min,ss_ target value, rather than a target AUC_τ,ss_, can be applied to TDM of TKIs in mRC.

### 6.2. Estimation of C_min,ss_ from a Blood Sample Collected during the Dosing Interval

**Figure 1:**
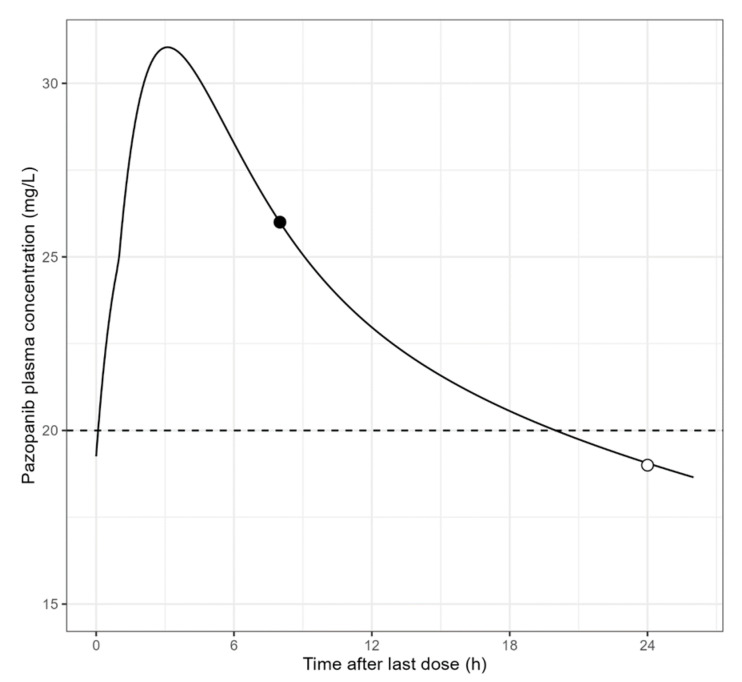
Analysis of pazopanib plasma concentration observed (●) at time 8 h after the last daily dose using mapbayr [60] in order to estimate C_min,ss_ (o) and to compare it with the target value (i.e., 20 mg/L).

On many occasions for practical purposes (e.g., outpatient and medical appointments differing from normal daily drug intake time), the actual C_min,ss_ cannot be determined. The measured level may be compared directly to the target C_min,ss_ only for TKIs with a particularly long half-life such as cabozantinib (around 55 h). For other TKIs, and even for cabozantinib, if the blood sample is obtained around C_max_ (shortly after drug intake), estimation of C_min,ss_ from the observed C would allow for a more accurate interpretation of TDM. This estimation may be performed by an *a posteriori* Bayesian approach implemented in several software tools. This consists in using a population PK model previously published for a drug to estimate the most likely individual PK parameters for the patient given her/his observed C. Figure 1 displays the result of a Bayesian analysis of pazopanib plasma concentration observed during the dosing interval to estimate C_min,ss_.

### 6.3. Pre-Analytical Aspects and Assay

Most TKIs, including the seven compounds used in mRC, are stable in blood at room temperature in the presence of light. Moreover, centrifugation of blood samples to obtain plasma can be delayed up to 72 h, facilitating sample shipment questions and delayed analysis. The HPLC or UPLC method coupled with MS-MS detection are generally used to quantify plasma TKI levels. The chromatographic step of the analysis is preceded by extracting the compounds from the plasma, usually performed by organic precipitation using either methanol or acetonitrile. Total (i.e., free and plasma bound) drug concentrations are thus measured.

### 6.4. Interpretation of TDM Results

The main result of TDM is the observed concentration value together, if necessary, with the estimated C_min,ss_ value. However, it is also necessary to provide bibliographic information allowing the physician to analyze this observed value together with clinical observation of the patient. As presented above, there is no universal threshold to classify exposure values in a toxic or efficacious zone. Therefore, the results should be interpreted in terms of over- or under-exposure compared to a median value or a range of observed values.

Below are the commentaries we propose for each of the seven TKIs.

Cabozantinib: the observed median residual concentration = 500 ng/mL (365.5–742.5) in a real-life study of 76 patients [31]; suggested that the efficiency threshold in this study is >530 ng/mL and the suggested toxicity threshold in this study is >620 ng/mL, to be compared with clinical tolerance.

Pazopanib: a residual concentration > 20.6 mg/L is necessary to obtain optimal efficiency [35].

Sorafenib: a residual concentration > 4.78 mg/L is associated with the development of major adverse effects [43]. Even if there is no validated efficiency threshold, we recommend a C_min_ concentration between 3.75 and 4.30 mg/L, the mean residual concentration observed in different studies being 4.2 mg/L [61]

Sunitinib: a total residual concentration (sunitinib + its active metabolite SU 12662) greater than 50 µg/L is required for optimum efficiency. This total residual concentration must be less than 100 µg/L to avoid the risk of toxicity [40]. During a daily schedule with no therapeutic window (37.5 mg/day continuously), this total concentration must be between 37.5 µg/L and 100 µg/L for optimum efficiency and to avoid potentially increased toxicity [61].

Regardless of the result, PK variability factors should be considered to interpret exposure values before proposing a dose adjustment. In the case of supratherapeutic exposure, different PK variability factors should be considered: CRP level [62], low albumin level, sarcopenia [63], and PK drug–drug interactions (CYP and P-gp inhibitors ^11^, complementary and alternative medicines). Finally, the influence of food on PK should not be underestimated for drugs such as cabozantinib and pazopanib [64]. With no explanations from previous factors, some genetic polymorphisms such as CYP3A4*22 [65], U GT1A1/9 [66,67,68] and P-gp/BCRP [44,69] might need to be explored since they can significantly decrease metabolism and/or enhance TKI bioavailability. In the case of subtherapeutic exposure, different parameters should be explored: adherence, PK DDI (CYP inducer [11], complementary and alternative medicines) and proton pump inhibitor uptake for tivozanib and pazopanib [11,66].

### 6.5. Combinations of Tyrosine Kinase Inhibitors with Immunotherapy

The current trend is to include TKIs in combination with immunotherapy, either against PD-1 or PD-L1, in treating mRC. The combinations of pembrolizumab–axitinib, nivolumab–cabozantinib, and lenvatinib–pembrolizumab have become the most widely accepted first-line therapy. The greater efficacy of these combinations vs. TKI monochemotherapy has been demonstrated by several phase 3 clinical trials showing a synergistic, or at least an additive, antitumoral effect. Moreover, given the very different toxicity profile of CPIs and TKIs, combining these two drugs at their standard dosages as used in monotherapy may be an option to consider. Indeed, axitinib in combination with pembrolizumab is given at its standard dose (i.e., a starting dose of 5 mg twice daily with the possibility of increasing up to 7 mg twice daily in the case of good arterial pressure tolerance) [70]. However, based on the results of the phase 1 trial combining cabozantinib and nivolumab which showed that 60 mg/day (the standard dose in monotherapy) of cabozantinib led to a higher rate of adverse events despite the absence of DLT, the RP2D chosen was 40 mg/day of cabozantinib [71]. Thus far, no report of PK/PD studies corresponding to these combinations has been published. However, in parallel to the generalization of combination therapy, it will be important to re-evaluate the PK/PD relationship described for TKIs in monotherapy, particularly those between TKI plasma concentrations and adverse events. If these future results are superimposed over those observed in monotherapy, it will prove that their dose-limiting toxicity is independent of CPIs. Where the adverse events vs. plasma concentration curve shifts to the left, it would provide evidence that an overall lower dose and lower target concentrations should be used with these combinations.

### 6.6. Plasma Protein Binding

TKIs, and particularly the seven compounds used in renal cancer, are highly bound to plasma proteins, serum albumin and α1-acid glycoprotein, with an unbound fraction (f_u_) around or even less than 1% for most of them (Table 1). According to a general concept in clinical pharmacology, confirmed by specific observations of imatinib, PK/PD relationships are closer when unbound plasma concentrations (C_u_) rather than total concentrations are considered, particularly when f_u_ presents large IIV. Indeed, binding to plasma protein represents a limiting factor for TKI diffusion within cells. Assays used for TDM make it possible to determine (total) plasma concentrations. Thus, plasma protein binding should be kept in mind for patients with a low level of plasma protein and/or treated by another drug capable of displacing TKI from plasma proteins. In these patients, plasma concentrations within the target range may be associated with higher C_u_, and consequently with a higher risk of adverse events.

### 6.7. Intrapatient Pharmacokinetic Variability

We have seen that one of the prerequisites for considering TDM for a drug is its limited intrapatient PK variability. Indeed, if such is not the case, a single observed plasma concentration may not be representative of drug exposure over a longer period. If intrapatient variability of each of the seven TKIs is indeed lower than their IIV, intrapatient PK is never negligible. Intrapatient variability has been estimated at 35% for pazopanib. Part of this variability is certainly linked to the food effect on pazopanib bioavailability. Simultaneous ingestion of food increases plasma exposure by approximately twofold, but to an extent this itself is dependent on lipid components. Finally, plasma exposure to pazopanib and sorafenib is known to decrease over the treatment course [52,72], especially after three months. This phenomenon can result in subtherapeutic exposure to TKI and insufficient anti-tumor effect. Using TDM would be particularly useful to restore therapeutic plasma exposure and therefore to optimize treatment time with pazopanib or sorafenib.

## 7. Conclusions

TDM represents a useful and complementary way to monitor patients with mRC treated by TKIs to individualize their dosage. There is a need for additional prospective clinical trials with PK-guided individualization, particularly within TKI–CPI combinations.

## Figures and Tables

**Table 1:** Pharmacokinetics of tyrosine kinase inhibitors prescribed in metastatic renal cancer.

	CL/F (L/h) IIV (%)	F (%)	Tmax (h)	Unbound Plasma Fraction (%)	Metabolic Pathways	Drug Transporters	Elimination Half-Life (h)	Ref
Sorafenib	8.13 (18)	ND	3	<1	CYP3A4, UGT1A9	MRP2	25–48	[13]
Sunitinib	46.4 (46)	ND	8.5	5	CYP3A4	BCRP	40–60	[14,15]
Pazopanib	0.458 (71)	21.5	3	<0.1	CYP3A4	P-gp; BCRP	27–36	[16,17]
Axitinib	32.3 (57)	58	3.9–6.0	1	CYP3A4, CYP1A2, CYP2C19, UGT1A1	P-gp; BCRP	2.5–6.1 (43)	[18]
Lenvatinib	6.5 (25.5)	ND	1–4	1–2	CYP3A4	P-gp; BCRP	20.6–34.3	[19]
Cabozantinib	2.2 (46)	ND	2–3	<1	CYP3A4, CYP2C9	MRP2, OAT3	120	[20,21]
Tivozanib	0.862 (men); 0.651 (women) (40.5)	ND	3	<1	CYP3A4, UGT?	-	108–121	[22]

CL/F, oral clearance; F, oral bioavailability; Tmax, time of maximum plasma concentration; ND, not determined; CYP, cytochrome P450; UGT, UDP-glucuronosyl transferase; MRP, multidrug resistance-associated protein; BCRP, breast cancer-resistant protein; P-gp, P-glycoprotein; OAT, organic anion transporter.
